# Tributyrin Supplementation Rescues Chronic–Binge Ethanol-Induced Oxidative Stress in the Gut–Lung Axis in Mice

**DOI:** 10.3390/antiox13040472

**Published:** 2024-04-17

**Authors:** Anthony Santilli, David Shapiro, Yingchun Han, Naseer Sangwan, Gail A. M. Cresci

**Affiliations:** 1 Department of Inflammation and Immunity, Lerner Research Institute, Cleveland, OH 44195, USA; santila@ccf.org (A.S.);; 2 Microbial Sequencing & Analytics Resource (MSAAR) Facility, Shared Laboratory Resources (SLR), Lerner Research Institute, Cleveland, OH 44195, USA; sangwan@ccf.org; 3 Cardiovascular and Metabolic Sciences, Lerner Research Institute, Cleveland Clinic, Cleveland, OH 44195, USA; 4 Cleveland Clinic Lerner College of Medicine, Case Western Reserve University, Cleveland, OH 44195, USA; 5 Department of Gastroenterology, Hepatology and Nutrition, Digestive Disease Institute, Cleveland, OH 44195, USA

**Keywords:** gut microbiome, butyrate, alcohol, lung inflammation, small intestine, gut immunity

## Abstract

Excessive alcohol consumption increases the severity and worsens outcomes of pulmonary infections, often due to oxidative stress and tissue damage. While the mechanism behind this relationship is multifaceted, recent evidence suggests ethanol-induced changes to the gut microbiome impact the gut–lung axis. To assess this, a chronic–binge ethanol feeding mouse model was used to determine how ethanol altered the gut microbiome, small intestinal epithelial barrier, and immune responses, as well as neutrophil abundance and oxidative stress in the lungs, and how supporting gut health with tributyrin supplementation during chronic–binge ethanol exposure affected these responses. We found that ethanol consumption altered gut bacterial taxa and metabolic processes, distorted small intestinal immune responses, and induced both bacteria and endotoxin translocation into the lymphatic and circulatory systems. These changes were associated with increased neutrophil (Ly6G) presence and markers of oxidative stress, lipocalin-2 and myeloperoxidase, in the lungs. Importantly, tributyrin supplementation during ethanol exposure rescued gut bacterial function (*p* < 0.05), small intestinal barrier integrity, and immune responses, as well as reducing both Ly6G mRNA (*p* < 0.05) and lipocalin-2 mRNA (*p* < 0.01) in the lungs. These data suggest ethanol-associated disruption of gut homeostasis influenced the health of the lungs, and that therapeutics supporting gut health may also support lung health.

## 1. Introduction

The gut microbiome comprises trillions of microbes, and in the past several years there has been an increasing appreciation regarding the important role the gut microbiome plays in supporting health. While there is intra-individual variability (alpha-diversity) in microbiome composition amongst healthy individuals, distinct dissimilarity (beta-diversity) between healthy individuals and those with various chronic diseases is noted [1]. At the phylum level, about 90% of the gut microbiome composition of a healthy individual comprises the Bacteroidetes and Firmicutes phyla. The gut microbiome functionally supports host health by aiding in the metabolism of nondigestible food components (e.g., fiber), mitigating pathogen invasion, and modulating the immune system [2]. The gut microbiome produces many beneficial metabolites for the host, including enzymes, vitamins, hormones, and short-chain fatty acids (SCFA; e.g., acetate, propionate, and butyrate). Butyrate is known to have many important biological functions, including supporting the integrity of the gut barrier and immune function [3,4].

Chronic heavy alcohol exposure is known to disrupt the gut microbiome’s composition and function, increase intestinal permeability, and activate systemic inflammatory pathways [1]. Chronic alcohol exposure reduces the abundance of Bacteroidetes and Firmicutes phyla, expands the Proteobacteria phylum, and lowers fecal SCFA levels, including that of butyrate [5]. In our prior studies in which mice exposed to chronic and chronic–binge ethanol were co-supplemented orally with tributyrin, a butyrate prodrug, or a butyrate-targeting synbiotic, supplemented mice were protected from proximal colon epithelial barrier disruption, as well as liver injury, inflammation, and oxidative stress, compared to non-supplemented ethanol-exposed mice [6,7,8,9]. Tributyrin is a structured lipid with three butyrate molecules esterified to the glycerol backbone. Upon oral ingestion, tributyrin is digested by pancreatic lipase to free up three butyrate molecules which are then absorbed throughout the intestine. The increased half-life of tributyrin compared to sodium butyrate makes it more appealing as a potential therapeutic [6].

The role of the gut microbiome in the health of other organ systems is being increasingly investigated. Among inter-organ relations, the gut–lung axis is less studied than either the gut–liver or the gut–brain axis. Chronic excessive alcohol exposure disrupts the body’s innate and adaptive immune system [10], and consequentially, individuals who consume excessive alcohol are at higher risk of acute lung injury and lung infections [11]. Chronic excessive alcohol exposure disrupts alveolar epithelial barrier integrity, reduces endogenous antioxidant levels (glutathione), and causes defects in alveolar macrophage function [11]. The microbiota of the gut and the lung influence the local immune system; however, there is also a potential for crosstalk between the gut and lungs. Impairment of the intestinal epithelial barrier by overgrowth of potentially pathogenic bacteria (gut dysbiosis) enables intact bacteria, bacterial fragments, or microbiome metabolites (e.g., SCFA) to translocate from the gut lumen into systemic circulation. This can be accomplished by entry of these components into the portal vein en route to the liver and then systemic circulation, and/or entry of these components into the draining mesenteric lymphatic system, thoracic duct, and then systemic circulation [12,13]. Eventually, both routes lead to the pulmonary system.

Chronic heavy alcohol consumption is known to induce oxidative stress and impair the gut mucosal barrier through its first metabolite acetaldehyde and induction of cytochrome P450 enzyme CYP2E1 [14]. In addition to supporting the integrity of the gut barrier and immunity, butyrate has been shown to have antioxidative effects [4]. There are limited data regarding the effects of butyrate supplementation during chronic–binge ethanol exposure on the small intestine and lung axis. Since we found tributyrin-supplemented mice exposed to chronic–binge ethanol had decreased oxidative stress, inflammation, and injury in the liver, here we tested our hypothesis that tributyrin supplementation during chronic–binge ethanol exposure would reduce oxidative stress in the lungs. Our data show that due to support of the gut microbiome, small intestinal epithelial barrier, and immune responses during chronic–binge ethanol exposure, tributyrin-supplemented mice also had less neutrophil abundance and oxidative stress in the lungs.

## 2. Materials and Methods

### 2.1. Chronic–Binge Ethanol Feeding Model

C57BL/6 female mice (10–11 weeks) were purchased from Jackson Laboratory (Bar Harbor, ME, USA). Mice were randomized to receive a chronic–binge ethanol Lieber DeCarli feeding model consisting of 10 days chronic ethanol (5% *v*/*v*) exposure and ±daily supplementation with saline or 5 mM tributyrin by oral gavage. Control animals were pair-fed an isocaloric diet with maltose dextrin substituted for ethanol ± 5 mM tributyrin or saline supplementation by oral gavage daily. On the 11th day, mice were administered an oral binge gavage of either 5 g/kg ethanol or maltose with either 2.5 mM tributyrin or saline as previously randomized. There were four groups in total: pair-fed saline (PF-S), pair-fed tributyrin (PF-TB), ethanol-fed saline (EF-S), and ethanol-fed tributyrin (EF-TB). Mice were anesthetized 6 h post-gavage, blood was collected from the inferior vena cava, and tissue (small intestine, cecum, lung) was dissected and stored for future analyses.

### 2.2. Shotgun Metagenomics Sequencing, Bioinformatics, and Statistical Analysis

gDNA from cecal contents was extracted using a Zymo Research Quick DNA Feca/Soil Microbiome MiniPrep Kit (Zymo Research, Irvine, CA, USA) following the manufacturer’s instructions. gDNA was quantified using a Nanodrop ND1000 Spectrophotometer (Thermo Fisher Scientific, Wilmington, DE, USA).

Quality control of the metagenomic reads was conducted as described previously [15]. Briefly, raw reads were processed for low-quality-based filtering using the Trimmomatic pipeline [16]. Host-derived reads were excluded by mapping the reads to the reference human genome (version GRCh38.p14) using BBMap software (sourceforge.net/projects/bbmap/ version 34.62). Quality trimmed reads were processed for taxonomic and functional profiling using Metaphlan2 [17] and Humann2 [18], respectively. Differential feature selection was performed using Fisher’s exact *t*-test. We assessed the statistical significance (*p* < 0.05) throughout, and whenever necessary, we adjusted *p*-values for multiple comparisons according to the Benjamini and Hochberg method to control the False Discovery Rate [19] while performing multiple testing on taxa and pathway abundances according to sample types.

Differential abundance test benchmarking was performed using the DAtest package (https://github.com/Russel88/DAtest/wiki/usage#typical-workflow version 2.8.0). Briefly, differentially abundant methods were compared with the False Discovery Rate (FDR), Area Under the (Receiver Operator) Curve (AUC), empirical power (Power), and the False Positive Rate (FPR). Based on the DAtest’s benchmarking, we selected LefSeq and analysis of variance (ANOVA) as the methods of choice to perform differential abundance analysis. We assessed the statistical significance (*p* < 0.05) throughout, and whenever necessary, we adjusted *p*-values for multiple comparisons according to the Benjamini and Hochberg method to control the False Discovery Rate [20]. Linear regression (parametric) and Wilcoxon (non-parametric) tests were performed on genera and species abundances against metadata variables using their base functions in R (version 4.1.2) [21].

### 2.3. Small Intestinal Isolation of Intraepithelial Lymphocytes, Lamina Propria Lymphocytes, and Intestinal Epithelial Cells

The small intestinal tract from the pylorus to the distal ileum was dissected following euthanasia. Single cells from intraepithelial lymphocytes (IELs), lamina propria lymphocytes (LPLs), and intestinal epithelial cells (IECs) were isolated and counted as described previously by Qiu et al. [22]. IELs and LPLs were stimulated with phorbol 12-myristate 13-acetate (PMA, 10 ng/mL)/ionomycin (1 µM) (MilliporeSigma, Darmstadt, Germany) for 4 h followed by 2 µM monensin (Calbiochem, San Diego, CA, USA) during the last 2 h of stimulation. The IECs were flash frozen and stored at −80 °C for future analyses.

### 2.4. Isolation of Lung Immune Cells

Following euthanasia, the lungs were perfused with cold PBS and the left lobe was dissected. A portion of the tissue was minced and then digested using a 1 mg/mL collagenase type 1 (Life Sciences Technologies, Grand Island, NY, USA) while incubating at 37 °C in an orbital shaker at 225 rpm for 30 min. The suspension was then filtered using a 70 µM strainer and immediately counted.

### 2.5. Flow Cytometry Analysis of IELs, LPLs, and Lung Immune Cells

Intestinal (IEL, LPL) and lung immune cells were washed and stained with either LIVE/DEAD Fixable Blue Dead Cell Stain (Invitrogen, Carlsbad, CA, USA) or LIVE/DEAD Fixable Near-IR Dead Cell Stain (Invitrogen, Carlsbad, CA, USA), respectively, and then fixed and permeabilized (True-Nuclear Transcription Factor Buffer Set, Biolegend, San Diego, CA, USA). Cells were washed and blocked to reduce nonspecific staining (FcR Blocking Reagent, Miltenyi Biotec, Gaithersburg, MD, USA). IELS and LPLs were stained with fluorescence-conjugated antibodies with anti-CD45-APCFire 810, anti-CD11c-BV650, anti-IL6-PerCPeFlour710, anti-CD3-FITC, anti-CD4-BUV805, and anti-CD8a-BUV615. Lung immune cells were stained with fluorescence-conjugated antibodies with anti-CD45-AF700, anti-SiglecF-BV421, anti-CD11b-BV605, and anti-Ly6G-PeCy7. Following staining, samples were washed 3 times, and cells were acquired using a Sony ID 7000 Spectral Cell Analyzer (San Jose, CA, USA). Analysis of the flow cytometry results was performed using FloJo ver. 10.8 (BD Life Sciences, Sparks, MD, USA).

### 2.6. Tissue RT-qPCR

Tissue was stored in RNAlater immediately after its dissection. Total RNA from the lung was extracted using an RNeasy Plus Universal Mini Kit (Qiagen, Hilden, Germany) following the manufacturer’s instructions. RNA was quantified using a Nanodrop ND1000 Spectrophotometer (Thermo Fisher Scientific, Wilmington, DE, USA), and 2 µg of total RNA was reverse transcribed using SuperScript IV VILIO (Invitrogen, Carlsbad, CA, USA). QuantStudio 5 (Applied Biosciences, Waltham, MA, USA) was used for Real Time PCR (RT-qPCR) amplification with a PowerUp SYBR Green Master Mix (Applied Biosciences, Waltham, MA, USA) and 1 µM primers (Table 1). The Comparative Threshold (CT) method was used to determine relative expression compared to the housekeeping gene glyceraldehyde 3-phosphate (GAPDH), and data are presented as mean ± standard deviation.

### 2.7. Western Blotting: Intestinal Epithelial Cells

Intestinal epithelial cells were suspended in lysis buffer (1% Triton X-100, 50 mM Tris-HCl pH 7.4, 150 mM NaCl, 1 mM EDTA pH 8, 0.1% Na-deoxycholate, 0.1% SDS) containing Pierce Protease and Phosphatase Inhibitor Mini Tablets (Thermo Fisher, Waltham, MA, USA) at a concentration of 8 × 10^6^ cells/100 µL, and lysates were prepared as previously reported [8]. Membranes were probed with anti-Junctional Adhesion Molecule A 1 (JAMA-1), anti-Zonula Occludens-1 (ZO-1), anti-PhosphoStat3 (pSTAT3), and anti-TotalStat3 (TSTAT3). Anti-Heat Shock Cognate Protein 70 (HSC70) was used as a loading control. Protein expression was visualized with enhanced chemiluminescence, and intensity was measured via densitometry using ImageJ software (National Institutes of Health (NIH), Bethesda, MD, USA, ver. 1.54i).

### 2.8. Plasma Endotoxin Quantification Using Limulus Amoebocyte Lysate (LAL) Assay

Blood samples were collected, and plasma was separated using techniques to avoid contamination using endotoxin-free materials. Glass tubes for sample dilutions were incubated at 250 °C for 40 min to eliminate residual bacteria or endotoxin. Samples were prepared as previously reported [23], and endotoxin concentration was measured using the Limulus Amebocyte Lysate Assay Kinetic-QCL Kit (Lonza, Walkersville, MD, USA). A standard curve was generated using *Escherichia coli* O55:B5 Endotoxin (Lonza, Walkersville, MD, USA). Plates were read and endotoxin concentration was quantified using SpectraMax Microplate Readers and SoftMax^®^ Pro 7.1.2 GxP Software Molecular Devices (San Jose, CA, USA).

### 2.9. Cecal Secretory IgA (SIgA) Enzyme-Linked Immunoassay (ELISA)

Supernatants from cecal contents were prepared and diluted as previously described [24], and IgA levels were assessed using a Mouse IgA uncoated ELISA Kit (Invitrogen, Waltham, MA, USA) according to the manufacturer’s instructions. The ELISA plate was read using a BioTek Synergy H1 Microplate Reader and BioTek Gen 5 software ver. 3.12 (Agilent Technologies, Santa Clara, CA, USA).

### 2.10. Lipocalin 2 (LCN2) and Myeloperoxidase (MPO) ELISA in Lung Tissue

Lung tissue samples were suspended in lysis buffer as described in the Mouse MPO ELISA Kit (HycultBiotech Inc, Wayne, PA, USA) at 10 mg/200 µL, and homogenized using a Branson Digital Sonifier 450 (Branson Ultrasonics, Danbury, CT, USA). Samples were then centrifuged at 1500× *g* at 4 °C for 15 min, and the supernatant was collected. Samples were diluted 1:1000 and both MPO and LCN2 levels were assessed using either a Mouse MPO ELISA Kit (HycultBiotech inc, Wayne, PA, USA) or a Quantkine ELISA Mouse Lipocalin-2/NGAL Immunoassay Kit (R&D Systems, Minneapolis, MN, USA), respectively, according to the manufacturer’s instructions. ELISA plates were read using a BioTek Synergy H1 Microplate Reader and BioTek Gen 5 software ver. 3.12 (Agilent Technologies, Santa Clara, CA, USA).

### 2.11. Plating of Peyer’s Patches on Enterococcosel Agar

Following small intestine dissection, Peyer’s patches were isolated using aseptic techniques and washed with sterile PBS. Peyer’s patches were suspended in sterile 0.17% Triton in PBS and lysed using a 1.5 mL microcentrifuge tube pestle. Samples were then serially diluted and plated on BBL Enterococcosel agar (BD Life Sciences, Sparks, MD, USA). Plates were then incubated aerobically at 37 °C for 48 h and CFUs were counted. 

### 2.12. Statistical Analysis

GraphPad Prism^®^ 10 ver. 10.1.2 (San Diego, CA, USA) was used for statistical analysis, and data are shown as the mean ± standard error of the mean. Two-tailed *t*-tests were used to identify statistical significance between treatment groups, and a *p* ≤ 0.05 was used as the threshold for statistical significance.

## 3. Results

### 3.1. Ethanol Exposure and Tributyrin Supplementation Alter the Gut Microbiome and Its Functional Pathways

Chronic alcohol consumption modifies gut microbial populations and metabolite production, impairs the intestinal barrier, and compromises immunity [25,26]. To characterize the microbiota and assess for changes to the mouse gut microbiome caused by chronic–binge ethanol treatment, we performed shotgun sequencing of bacterial gDNA isolated from cecal contents.

**Figure 1:**
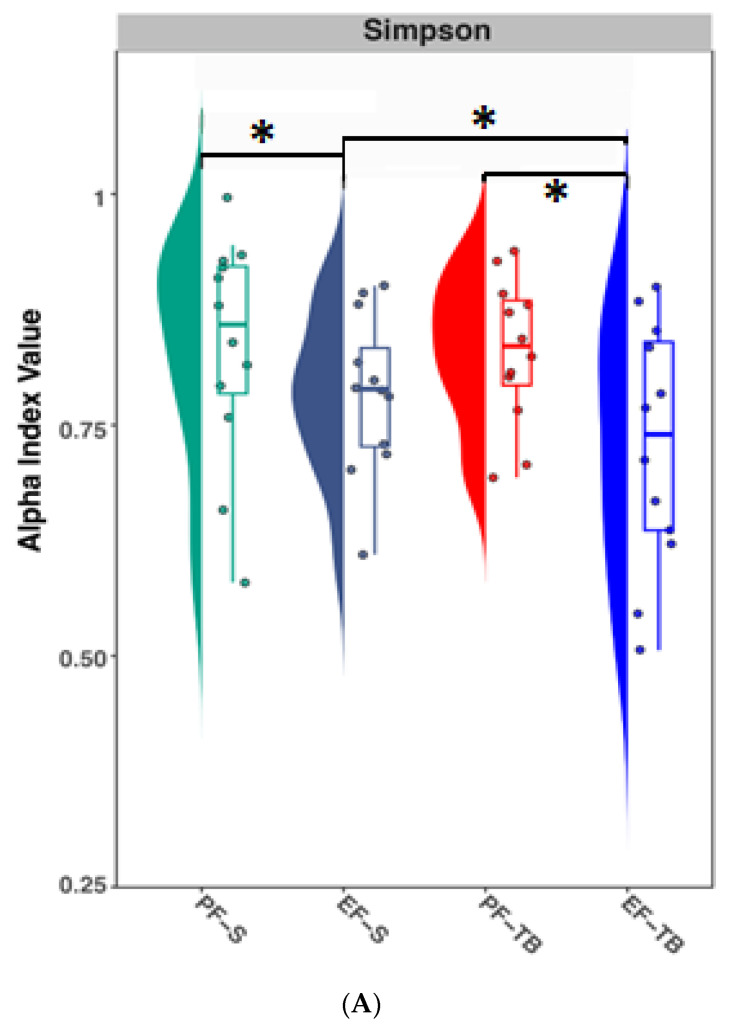

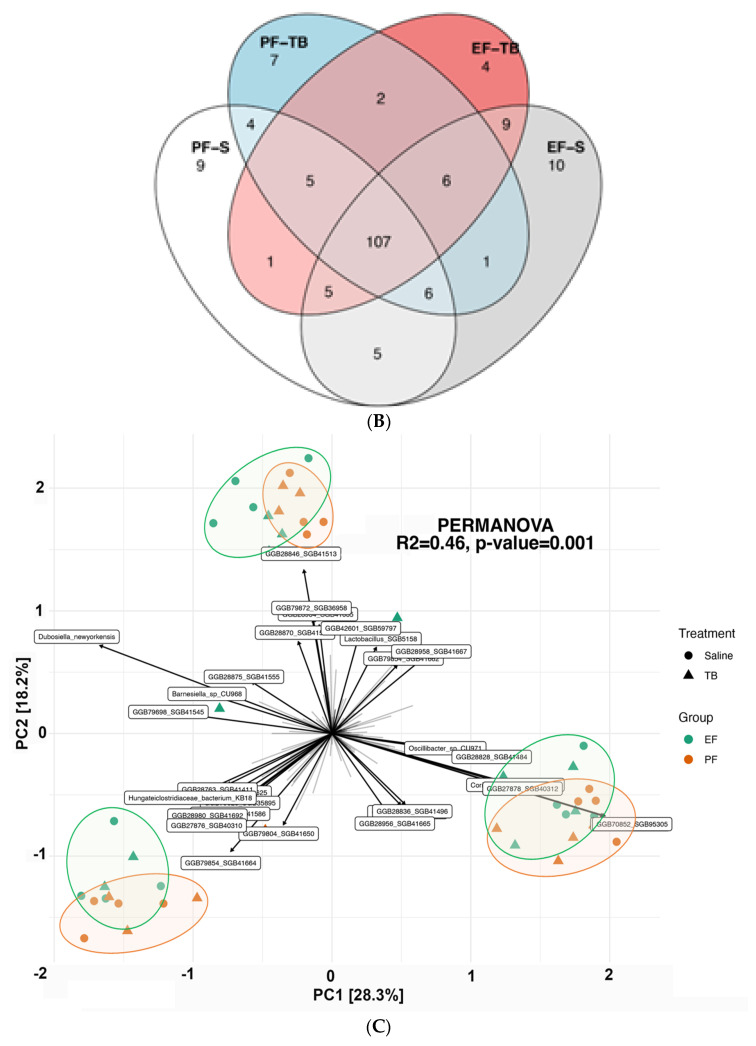

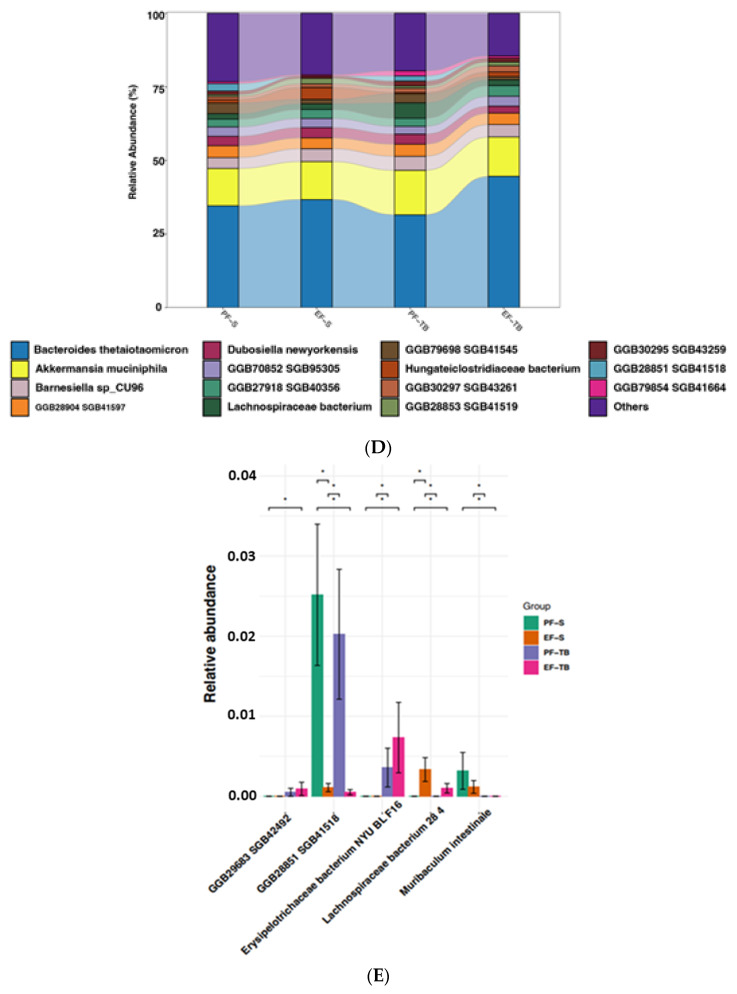
Effects of chronic–binge ethanol exposure ± tributyrin on the mouse gut bacteria taxonomy. (**A**) Alpha-diversity of cecal microbiome calculated using Simpson diversity index. (**B**) Venn diagram demonstrating species taxa separation and overlap among treatment groups. (**C**) Principal coordinate analysis plots generated using Bray–Curtis dissimilarity with PERMANOVA analysis. Ellipses are used to visually highlight differences between ethanol (green) and control (orange) diets and do not represent statistical analysis. (**D**) Stacked graph showing the relative abundance of top taxa at species level present in cecal contents. (**E**) Relative abundance of species showing significant differences between treatment groups with LEfSe statistical analysis. PF-S: control diet + saline gavage; EF-S: ethanol diet + saline gavage; PF-TB: control diet + tributyrin gavage; EF-TB: ethanol diet + tributyrin gavage; * *p* ≤ 0.05.

Measurement of diversity and evenness of bacterial species were shown to be decreased in ethanol-fed mice compared to pair-fed mice with both saline and tributyrin supplementation (Figure 1A; Simpson indexes *p* < 0.041 and *p* < 0.038, respectively). In comparing the Simpson index of each ethanol-treated group, mice supplemented with tributyrin had significantly lower diversity and evenness compared to mice supplemented with saline (Simpson index *p* = 0.021; Figure 1A). To quantify the dissimilarity between groups (beta-diversity), Bray–Curtis analysis was performed and Venn diagram and principal coordinate analysis (PCoA) plots were drawn. Depicted in the Venn diagram, a total of 107 species were shared across all groups; however, each mouse group had significant species based on treatment and supplementation, with the EF-S group having the highest number of unique species (*n* = 10), followed by PF-S (*n* = 9), PF-TB (*n* = 7), and EF-TB (*n* = 4) (Figure 1B; *p* < 0.05). PCoA analysis shows formation of three distinct clusters with significant dissimilarity between ethanol (green) and pair-fed (orange) groups (Figure 1C; *p* = 0.001). Relative abundance analysis identified bacterial species that drive diversity across treatment groups. *Bacteroides thetaiotaomicron*, a gut commensal, was the most prevalent species and was seemingly elevated with ethanol exposure, with the ethanol–tributyrin group exhibiting the highest abundance (Figure 1D). Both pair-fed groups also showed a higher abundance of several unknown species (GGB7968 SGB41545, GGB28851 SGB41518, and GGB79854 SGB41664; Figure 1D) compared to ethanol-fed mice. Five species, two of which are unknown, showed significant differences in relative abundance across the four treatment groups. *Erysipelotrichaceae bacterium* NYU-BL-F16 was induced with tributyrin supplementation (*p* < 0.05), *Lachnospiraceae bacterium* 28-4 was significantly increased in the EF-S vs. PF-S groups (*p* < 0.05) and lowered in the EF-TB group, and tributyrin lowered *Muribaculum intestinalie* in both pair- and ethanol-fed mice (*p* < 0.05) (Figure 1E).

**Figure 2:**
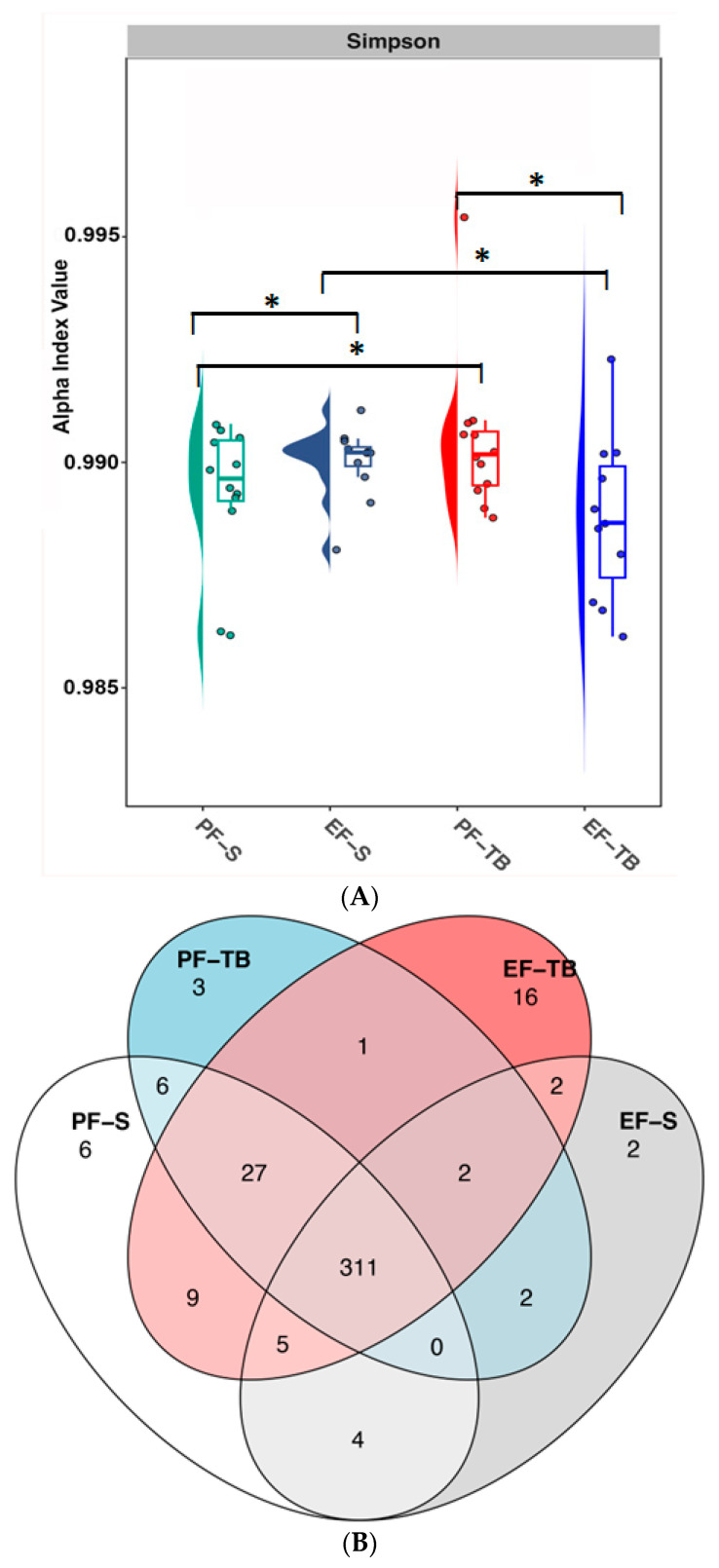

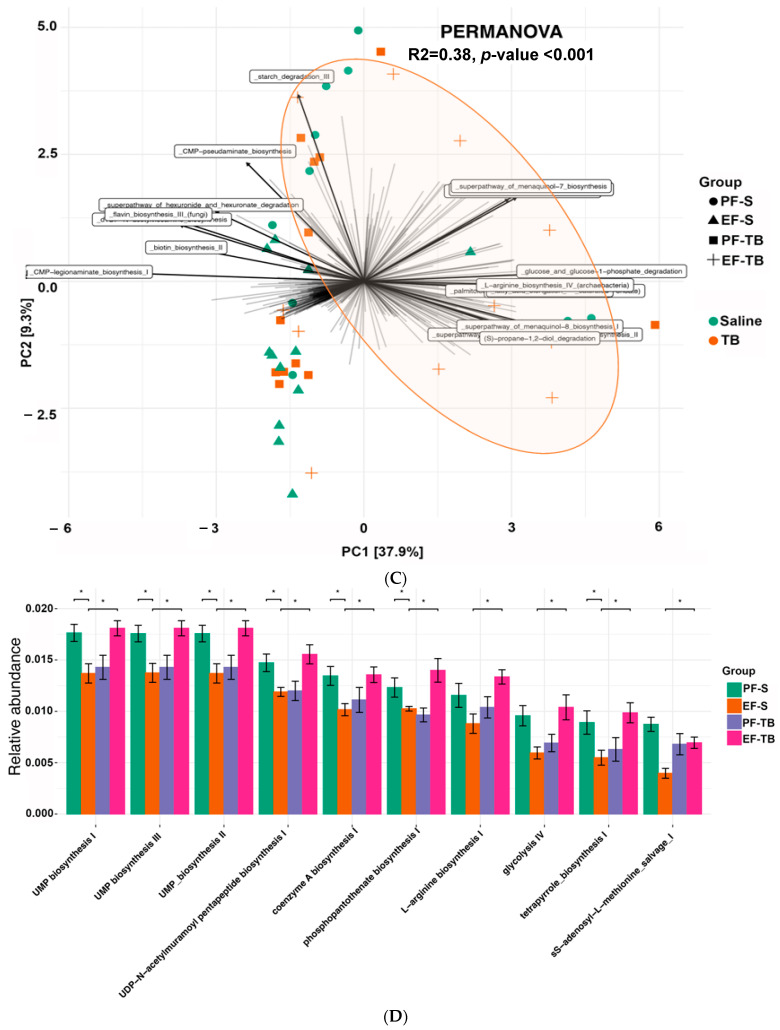
Tributyrin supplementation mitigates ethanol’s effects on gut microbiome function. (**A**) Alpha-diversity of cecal microbiome metabolic pathways calculated using Simpson diversity index. (**B**) Venn diagram demonstrating cecal bacteria metabolic pathway overlap between treatment groups. (**C**) Principal coordinate analysis plots generated using Bray–Curtis dissimilarity with PERMANOVA analyses. Orange-colored ellipse visually highlights the separation of the EF-TB group but does not represent statistical analysis. (**D**) Relative abundance graphs of cecal bacteria metabolic pathways showing significant differences between treatment groups with ANOVA testing. PF-S: control diet + saline; EF-S: ethanol diet + saline; PF-TB: control diet + tributyrin; EF-TB: ethanol diet + tributyrin; * *p* ≤ 0.05.

Shotgun sequencing of cecal bacteria also revealed gene changes to metabolic pathways of bacteria induced by chronic–binge ethanol exposure. The Simpson alpha-diversity index of gene pathways was reduced in the EF-TB group compared to all other groups (Figure 2A). To quantify the functional dissimilarity between groups (beta-diversity), Bray–Curtis analysis was performed and Venn diagram and principal coordinate analysis (PCoA) plots were drawn. The cecal bacterial gDNA in the EF-TB group displayed the most unique metabolic pathways (*n* = 16), as well as a large overlap of 27 pathways shared with both PF groups, but distinct from the EF-S group, suggesting tributyrin supplementation during ethanol exposure restored these pathways to those of PF mice (Figure 2B). PCoA plots of Bray–Curtis dissimilarity (Figure 2C) revealed distinct gene clustering between each group, demonstrating each exposure and/or supplementation altered the function of bacteria in a unique manner. Specifically, the EF-TB group clustered separately from the other groups (Figure 2C; orange crosses highlighted in ellipse). Assessment of the relative abundance of the top ten metabolic pathways showed they were all reduced in the EF-S group, and this trend was recovered with tributyrin supplementation. Several pathways are involved in the mitigation of oxidative damage, such as Coenzyme A (CoA) production [27] (CoA and phosphopentathonate biosynthesis), tetrapyrrole biosynthesis [28,29,30], S-adenosyl-l-methionine (SAM) salvage [30], and L-arginine biosynthesis [31,32] (Figure 2D). Pathways for peptidoglycan synthesis and modification, such as uridine monophosphate (UMP) biosynthesis I, II, and III, uridine diphosphate N-acetylmuramyl pentapeptide synthesis, and glycolysis, were also disturbed in the same manner (Figure 2D).

### 3.2. Ethanol Affects Intestinal Epithelial Immune Responses

**Figure 3:**
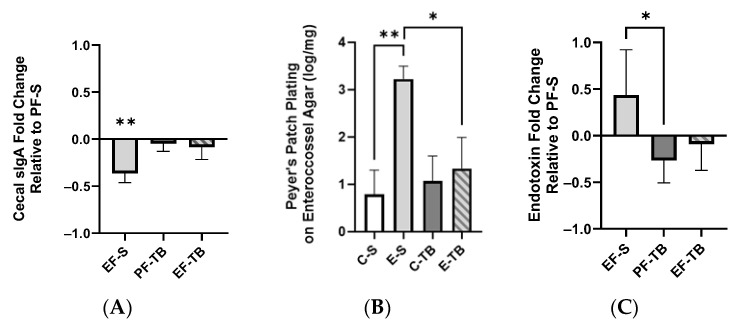
Ethanol exposure depletion of cecal SIgA and induction of bacterial translocation into the lymphatic and circulatory systems. ** denotes a significant decrease in the EF-S group vs. PF-S. (**A**) Concentration of SIgA in mouse cecal contents presented as fold of the control (PF-S). (**B**) *Enterococci* bacterial growth in small intestinal Peyer’s patches. (**C**) Plasma endotoxin concentration. Fold of the control (PF-S). Treatment groups include 6–8 mice. PF-S: control diet + saline; EF-S: ethanol diet + saline; PF-TB: control diet + tributyrin; EF-TB: ethanol diet + tributyrin; * *p* < 0.05; ** *p* < 0.01.

Disruptions to the gut microbiome in mice by chronic alcohol consumption and antibiotic treatment have been reported to decrease secretory IgA (SIgA) levels in cecal contents [24] and feces [33], respectively. SIgA serves as the first line of immune defense in protecting the intestinal epithelium from enteric toxins and pathogens. SIgA is synthesized by B cells upon antigen presentation by dendritic cells, and is secreted into the intestinal lumen to prevent microbes from penetrating the mucosal barrier and interfacing with intestinal epithelial cells [34,35]. We found that mice exposed to chronic–binge ethanol and saline displayed significantly reduced cecal SIgA levels relative to pair-fed saline mice, and tributyrin supplementation mitigated this but not significantly (Figure 3A; *p* = 0.006) A deficiency in this first-line mucosal immune defense allows for luminal bacteria to translocate across the epithelial barrier. Peyer’s patches are part of the gut-associated lymphoid tissue (GALT) and contain many dendritic cells, macrophages, and lymphocytes that help maintain a balanced gut microbiota and keep pathogens at bay. We found significantly increased *Enterococci* in the Peyer’s patches of EF-S mice, which was resolved with tributyrin co-supplementation so that EF-TB group counts were similar to those of pair-fed mice (Figure 3B).

Since ethanol provoked gut dysbiosis and increased the presence of bacteria in the Peyer’s patches, we assessed plasma endotoxin levels as a measurement of impaired intestinal barrier and a resulting systemic proinflammatory effect. Plasma endotoxin was elevated in the EF-S group, and tributyrin supplementation normalized plasma endotoxin to levels similar to those in the pair-fed groups (Figure 3C; *p =* 0.013).

### 3.3. Tributyrin Supplementation Bolsters Immune and Barrier Functions Both at the Intestinal Epithelium and within the Lamina Propria

**Figure 4:**
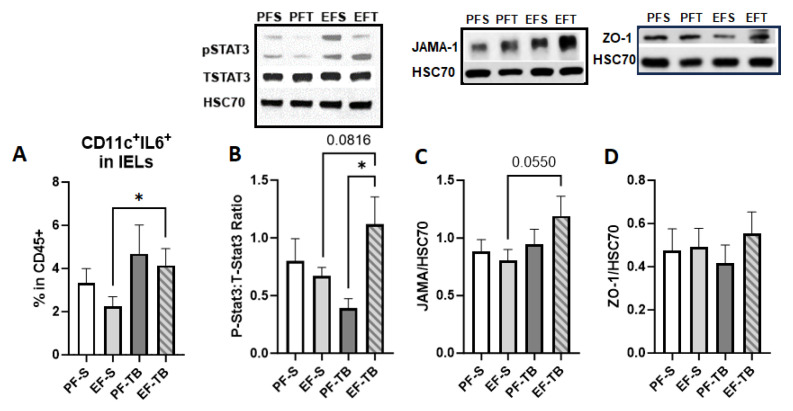
Tributyrin supplementation bolsters both the epithelial barrier and immunity. (**A**) %CD11c^+^IL-6^+^ cells isolated from IELs. Gating strategy for flow analysis is described in Figure S1. (**B**) Ratio of PhosphoStat3 to TotalStat-3 expression in IECs isolated from the small intestine. (**C**) JAMA protein expression in IECs isolated from the small intestine. (**D**) ZO-1 protein expression in IECs isolated from the small intestine. Treatment groups contain 5–8 replicates. PF-S: control diet + saline; EF-S: ethanol diet + saline; PF-TB: control diet + tributyrin; EF-TB: ethanol diet + tributyrin; * *p* < 0.05.

Proinflammatory mediators traveling from the intestinal lumen into systemic circulation must first penetrate the epithelial barrier and circumvent immune responses in both the intestinal epithelium and lamina propria. As butyrate is known to support the intestinal barrier and its immune responses, we assessed for IEL dendritic cells and found an elevation in IEL host defense via increased percentage of CD11c^+^IL-6^+^ dendritic cells in EF-TB compared to EF-S mice (Figure 4A). This response coincided with increased pSTAT3 in the EF-TB group vs. the PF-TB group, and a trend towards an increase from the EF-S group (Figure 4B). IL-6 at low levels has been shown to promote the phosphorylation of Stat3 in epithelial cells, which then promotes repair of the mucosal barrier by inducing B-cell differentiation needed for IgA production [36]. JAMA-1 and ZO-1 are two of many proteins that comprise the intestinal epithelial barrier complex. JAMA-1 spans the paracellular space and helps to seal the gap between epithelial cells, and ZO-1 serves as an anchor protein to multiple other junctional proteins, and when either protein is diminished the epithelial barrier’s integrity may be compromised. We found an induction of JAMA-1 in the small intestinal IEC of EF-TB mice, but there were no differences in ZO-1 expression between groups (Figure 4C,D).

**Figure 5:**
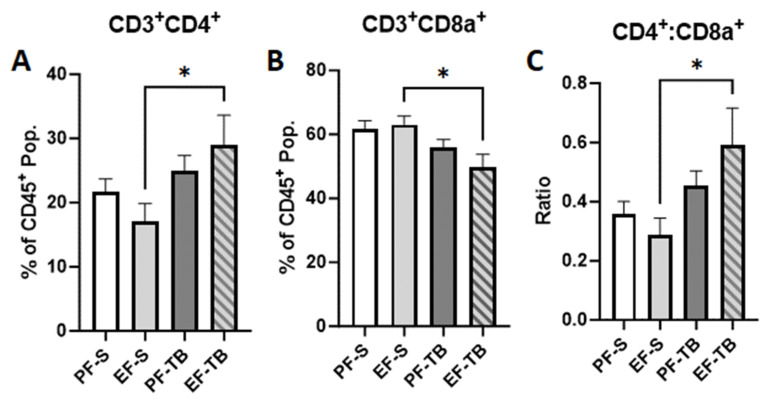
Percentage of CD4+ and CD8a+ T-cells in the lamina propria of the small intestine. (**A**) % CD3^+^CD4^+^ cells isolated from LPLs. (**B**) % CD3^+^CD8a^+^ cells isolated from LPLs. (**C**) Ratio of CD3^+^CD4^+^ to CD3^+^CD8a^+^ cells isolated from LPLs. Gating strategy for flow analysis is described in Figure S2. Treatment groups contain 7–8 replicates. PF-S: control diet + saline; EF-S: ethanol diet + saline; PF-TB: control diet + tributyrin; EF-TB: ethanol diet + tributyrin; * *p* < 0.05.

If intestinal epithelial defenses are breached, bacteria or bacterial byproducts can traverse through the lamina propria and its immune repertoire prior to entering circulation. Assessing for T cells within the lamina propria, we found an elevated percentage of CD3^+^CD4^+^ T helper/inducer cells (Figure 5A) and a reduced percentage of CD3^+^CD8a^+^ cytotoxic/suppressor T cells (Figure 5B) in the EF-TB group compared to the EF-S group. This resulted in an overall increased CD4:CD8 ratio in EF-TB mice compared to EF-S mice (Figure 5C), which is congruent with healthy immunity.

### 3.4. Tributyrin Supplementation Mitigates Ethanol-Induced Neutrophil Presence and Markers of Oxidative Stress in the Lungs

Bacteria or bacterial antigens in systemic circulation, arising from either the gut lymphatic system or vasculature, have been shown to induce immune cell infiltration and proinflammatory responses in outlying organ systems such as the hepatic, pulmonary, and nervous systems [12,13,37]. Since we found chronic–binge ethanol exposure altered the gut microbiome and impaired the gut barrier and immune responses which resulted in more bacteria in the Peyer’s patches and endotoxin in circulation, we assessed how these changes affected the lungs. We hypothesized that tributyrin’s protection of these effects in the gut would also impart a protective effect against immune infiltration and oxidative stress in the lungs.

**Figure 6:**
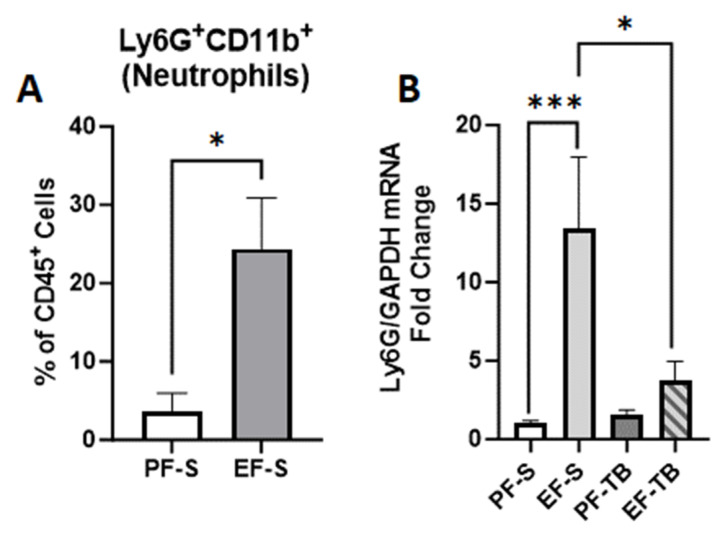
Neutrophil presence in the lungs. (**A**) % Ly6G^+^CD11b^+^ cells (neutrophils) isolated from lung homogenate. (**B**) Ly6G mRNA expression in lung tissue. Fold changes relative to PF-S group. Gating strategy for flow cytometry is described in Figure S3. Treatment groups contain 4–8 replicates. PF-S: control diet + saline gavage; EF-S: 5% ethanol diet + saline gavage; PF-TB: control diet + tributyrin gavage; EF-TB: 5% ethanol diet + tributyrin gavage; * *p* < 0.05; *** *p* < 0.0001.

We found an accumulation of the percentage of Ly6G+CD11b+ neutrophils in the EF-S mice compared to the PF-S mice by flow cytometry (Figure 6A; *p* = 0.048). Lung tissue Ly6G mRNA expression was also increased in EF-S mice, and tributyrin supplementation reduced this effect (Figure 6B; *p =* 0.0001). As activation of neutrophils can induce oxidative stress, we assessed for two markers, MPO and LCN2.

**Figure 7:**
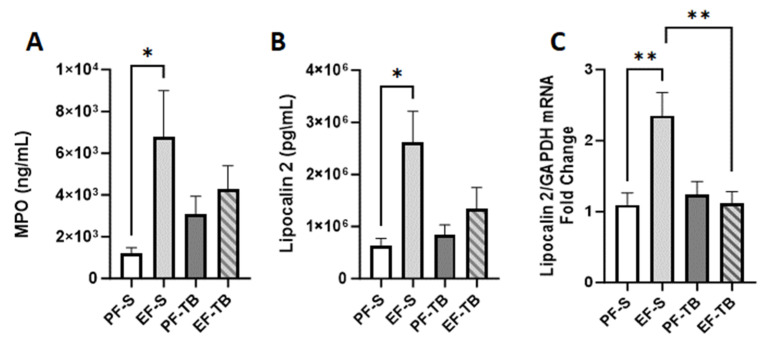
Markers of oxidative stress, MPO and LCN2, in lung. (**A**) MPO protein levels in lung tissue via ELISA. (**B**) LCN2 mRNA expression via qRT-PCR in lung tissue. Fold change is relative to PF-S. (**C**) LCN2 protein levels in lung tissue via ELISA. Treatment groups contain 7–8 replicates. PF-S: control diet + saline; EF-S:ethanol diet + saline; PF-TB: control diet + tributyrin; EF-TB: ethanol diet + tributyrin; * *p* <0.05; ** *p* < 0.01.

Activated neutrophils can induce oxidative stress responses. As neutrophils in lungs were elevated with chronic–binge ethanol exposure, we tested for markers of oxidative stress. MPO catalyzes the production of powerful pro-oxidant species capable of damaging surrounding tissue [38]. We found that the MPO protein level was elevated in lung tissue in EF-S compared to PF-S mice (Figure 7A; *p* = 0.0466). LCN2 sequesters bacterial siderophores to starve them of iron and is involved in mediation of oxidative stress [39,40,41]. LCN2 mRNA and protein expression were induced in EF-S mice, and tributyrin supplementation reduced the mRNA expression of LCN2 in ethanol-exposed mice (Figure 7B,C).

## 4. Discussion

Here, we show that mice exposed to chronic–binge ethanol have alterations in gut microbiome composition and function, decreased intestinal barrier defenses, skewed immune defenses, and increased bacteria and endotoxin in the GALT and systemic circulation, respectively, and this coincided with increased neutrophil presence and oxidative stress response in the lungs. Importantly, supplementation with tributyrin not only supported gut health disrupted by ethanol, but it also rescued the negative effects of ethanol in the lungs.

Analysis of the cecal microbiome showed that ethanol consumption significantly decreased species alpha-diversity (intra-individual variability), and this was further decreased with tributyrin supplementation. Significant dissimilarity (beta-diversity) between groups was also noted, driven by specific taxa. A portion of the microbiome was unidentified, and several unknown species of the Firmicutes phylum seemingly drove the marker of diversity assessed. Taxa clustering emerged based on both diet and treatment, indicating that while both factors are capable of shifting microbial composition, several species changed based on ethanol consumption alone. Bacteria belonging to the *Lachnospiraceae* family are known SCFA producers. The species elevated in both ethanol-exposed groups, *Lachnospiraceae* bacterium 28–4, has been predicted to possess pathways involved in butyrate synthesis [42]. Tributyrin supplementation decreased bacterial diversity and evenness, which is likely due to its alteration of specific taxa. One taxon induced by tributyrin was *B. thetaiotaomicron*, one of the most abundant commensal microbes that is involved with the metabolism of complex carbohydrates and maturation of the host immune system [43]. *Erysipelotrichaceae bacterium* NYU-BL-F16 also expanded with tributyrin supplementation [44]. While poorly characterized, its presence has been associated with a reduction in host metabolic pathways involved in diet-induced obesity [44]. In both the pair- and ethanol-fed groups, tributyrin supplementation decreased the relative abundance of *Muribaculum intestinale* (*MI*). This taxon produces a cardiolipin known to initiate an innate immune response and production of proinflammatory cytokines such as TNF-a, IL-6, and IL-23 [45]. Overall, tributyrin supplementation during ethanol exposure expanded gut microbiome species that contribute to the production of SCFA and support of the immune system compared to the results in mice only exposed to ethanol, and this may have contributed to maintenance of the immune effects noted in the hosts’ cells.

Shotgun metagenomic sequencing provides information regarding the function of the bacteria through identification of gene pathways. Ethanol exposure restricted several metabolic pathways of the cecal bacteria. Importantly, all top reduced pathways by ethanol exposure were recovered with tributyrin supplementation. Several altered metabolic pathways are noteworthy for their role in mediating immunity and mitigating oxidative stress. Coenzyme A and its precursor pantothenate mitigate reactive oxygen species through the formation of disulfide bonds between the thiol functional group and sulfur-containing residues of various proteins [27]. L-arginine is involved in the production of nitric oxide, and disruption in L-arginine metabolism is associated with immune-mediated or infectious diseases [31,32]. Tetrapyrroles form the basis of a variety of metal chelating compounds that display antioxidant properties [28,29,46] and are regulated by S-adenosyl-l-methionine (SAM) enzymes [30]. Additionally, SAM and SAM enzymes regulate several processes involved with oxidative stress management, such as the production of glutathione and regulation of tetrapyrrole synthesis [30,47,48]. Taken together, the loss in each of these individual pathways with ethanol exposure alone indicates an impairment of the bacteria in mitigating oxidative damage in their local environment. Such damage not only affects microbial health but can also contribute to oxidative stress, leading to inflammation, tissue damage, and altered immune responses [49,50]. Interestingly, a loss occurred in pathways involved in the synthesis of peptidoglycan, a primary component of the gram-positive bacteria cell wall. Moreover, it was gram-positive bacteria (Enterococci) that were cultured from the Peyer’s patches of EF-S mice. Peptidoglycan recognition by host immune cells is necessary to mount an immune response, and reduction and alteration to peptidoglycan structures have been suggested to allow these bacteria to circumvent such responses and contribute to their pathogenesis [51,52]. Taken together, tributyrin supplementation rescued specific gut microbiome taxa and their functions needed to combat the negative effects ethanol imposes on immunity and oxidative stress.

The changes induced in gut microbiome taxa and function by ethanol impacted immune functions in both the small intestine and lungs. Firstly, there was a depletion in cecal SIgA in the EF-S group. SIgA is found at low levels under normal conditions but is up-regulated upon presentation of antigen by dendritic cells to B cells. SIgA binds to bacteria, entrapping them so they are unable to interact with or penetrate the intestinal epithelium [34,35]. Bound bacteria can also be transported by M cells into Peyer’s patches in the small intestine or lymphatic follicles in the colon as a mechanism to initiate an immune response [33,34,35,53]. Ethanol-induced loss of SIgA opens a route for bacteria or antigen to reach the intestinal epithelium and translocate into systemic circulation. Indeed, plasma endotoxin concentration was elevated in the EF-S group, but this was normalized with tributyrin supplementation. While the portal vein is typically thought of as the primary route for pathogens or their associated molecular patterns originating from the gut to enter circulation, the lymphatic tissue that drains the gut has also been implicated as an alternative pathway [13,54]. Bacteria, endotoxin, or activated immune components that collect in lymphatic tissue in both the small and large intestines drain into the mesenteric lymph nodes and reach the thoracic duct. The contents of the thoracic duct then enter primary circulation via the subclavian vein. Taken together, compromise in gut luminal IgA could be an initial entryway for bacteria or bacterial endotoxins to reach the lungs.

Heavy alcohol consumption has been widely observed to impact pulmonary immunity and infections. Our results corroborate prior work from Arteel et. al., who have shown that the Lieber DeCarli chronic–binge model induces neutrophil accumulation in both bronchoalveolar lavage fluid and lung tissue, leading to a mild inflammatory response [55]. We also found increased LCN2 and MPO proteins, released by neutrophils in response to bacteria and their proinflammatory compounds. We posit disrupted intestinal homeostasis by ethanol and skewed oxidative stress defenses in both the gut microbiome and host cells, and impaired mucosal barrier and immune function open a path for bacteria and endotoxins to enter the lung to drive an immune response characterized by the accumulation of neutrophils and release of MPO and LCN2. Under normal conditions, such a response is necessary for host defense, but with persistent presence of these proteins, oxidative tissue damage in the lungs and ultimately pathology could result. For instance, the release of MPO and the resulting enzymatic products displays potent bactericidal properties, but this effect is not exclusive to pathogens and, if prolonged, can damage host tissue [38,56]. Lipocalin 2 is produced by neutrophils in response to the presence of bacteria and endotoxins and sequesters bacterial siderophores, depriving them of iron necessary for their growth. Through this ability to capture and stockpile iron, LCN2 has been shown to both induce and protect against oxidative stress depending on the context [39].

As a potential therapeutic to restore and bolster gut immune and barrier function disrupted by ethanol exposure, tributyrin co-supplementation was shown to support the gut–lung axis. Tributyrin supplementation resolved the expansion of *Muribaculum intestinali* and recovered bacterial metabolic pathways important for the regulation of oxidative stress in the small intestine. Additionally, enhancement of both the mucosal and epithelial barriers and immune responses in both the epithelium and lamina propria occurred. Such protection reduced both bacterial presence in Peyer’s patches and endotoxin in circulation, and in turn lowered neutrophil populations and MPO and LCN2 in the lung. Taken together, these data support our hypothesis that neutrophil activation in the lung is linked with ethanol-induced disturbances to the small intestine. These studies do have limitations in that they are associative findings and not cause and effect, necessitating futher studies. Also, we utilized a pre-clinical mouse model that replicates hepatic phenomena as human alcoholic hepatitis, with controlled environments and co-supplementation of tributyrin with ethanol exposure. Whether tributyrin protection would occur as a treatment in humans with alcoholic hepatitis and pulmonary compromise is uncertain.

## 5. Conclusions

Chronic–binge ethanol exposure disrupts the gut–lung axis. While the association between alcohol consumption and oxidative damage in the lungs is well documented, this work supports the notion that these processes are driven by ethanol-induced damage to the gut microbiome’s composition and function, the small intestine mucosal barrier, and immunity. Importantly, tributyrin supplementation supported intestinal immune and barrier function, as well as reducing neutrophils and oxidative stress-related proteins in the lungs. These findings highlight the unique ability of intestinal health to influence the lung. Further investigations into how the gut–lung crosstalk occurs for future novel treatments of alcohol-related pulmonary conditions are warranted.

## Figures and Tables

**Table 1:** RT-qPCR primers.

Target	Primer	Sequence
Lymphocyte Antigen 6 Complex Locus G6D (Ly6G) NM_001310438	Ly6G F	TGC GTT GCT CTG GAG ATA GA
	Ly6G R	CAG AGT AGT GGG GCA GAT GG
Lipocalin 2 NM-008491	LCN2 F	TGG CCC TGA GTG TCA TGT G
	LCN2 R	CTC TTG TAG CTC ATA GAT GGT GC

## Data Availability

Data supporting the reported results can be requested by writing to the corresponding author.

## Supplementary Materials

The following supporting information can be downloaded at https://www.mdpi.com/article/10.3390/antiox13040472/s1: Figure S1. Gating strategy for CD11c^+^IL-6^+^ cells; Figure S2. Gating strategy for CD3^+^CD4^+^ and CD3^+^CD8a^+^ cells; Figure S3. Gating strategy for Ly6G^+^CD11b^+^ cells.
