# Supplementary material for: Tributyrin Supplementation Rescues Chronic–Binge Ethanol-Induced Oxidative Stress in the Gut–Lung Axis in Mice
Source: Antioxidants (Basel). 2024 Apr 17;13(4):472. doi: 10.3390/antiox13040472 (PMC11047693; doi:10.3390/antiox13040472)
Supplement: Supplementary file 1 [file antioxidants-13-00472-s001.zip › antioxidants-2953757-supplementary.pdf]

# Tributyrin supplementation alleviates chronic-binge ethanol induced oxidative stress in the gut-lung axis

Supplementary Materials

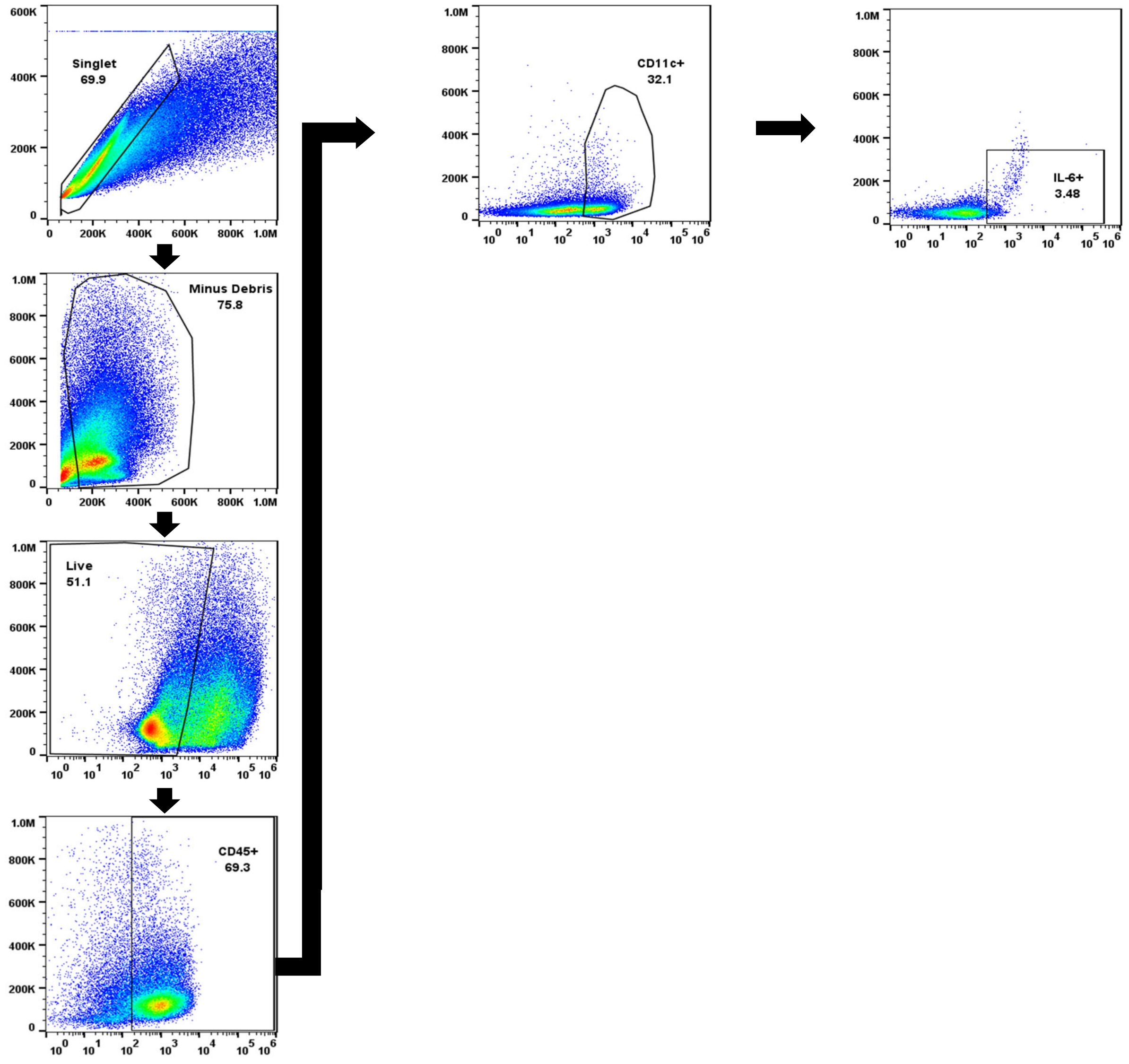


**Figure S1.** Flow gating strategy for the identification of CD11c$^+$IL-6$^+$ cells in intraepithelial lymphocytes.

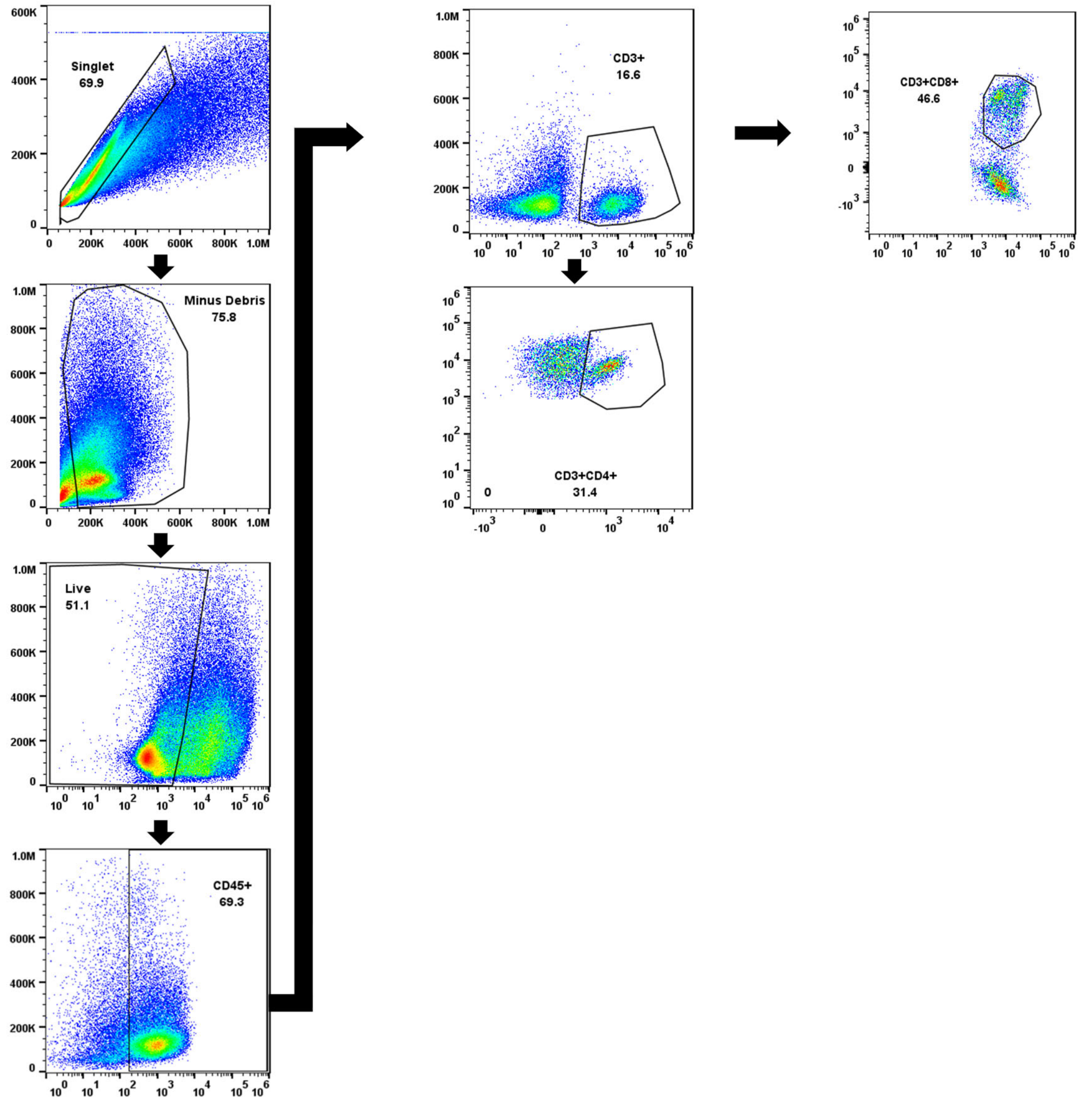


**Figure S2.** Flow gating strategy for the identification of CD3$^+$CD4$^+$ and CD3$^+$CD8a$^+$ cells in lamina propria lymphocytes.

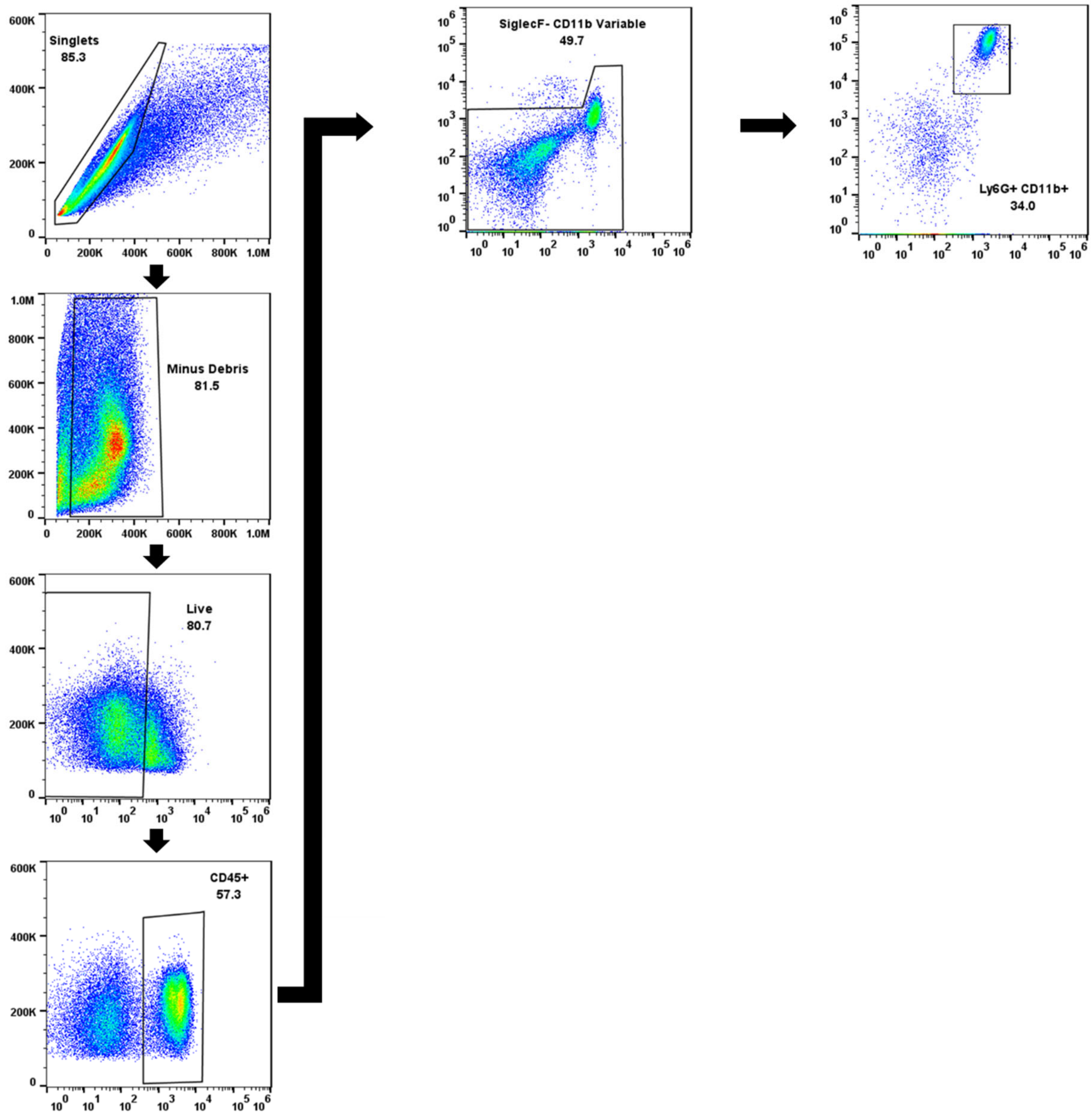


**Figure S3.** Flow gating strategy for the identification of Ly6G⁺CD11b⁺ cells from lung cells.
